# Emerging magnetic order in platinum atomic contacts and chains

**DOI:** 10.1038/ncomms7172

**Published:** 2015-02-04

**Authors:** Florian Strigl, Christopher Espy, Maximilian Bückle, Elke Scheer, Torsten Pietsch

**Affiliations:** 1 Department of Physics, University of Konstanz, Universitätsstraße 10, D-78464 Konstanz, Germany

## Abstract

The development of atomic-scale structures revealing novel transport phenomena is a major goal of nanotechnology. Examples include chains of atoms that form while stretching a transition metal contact or the predicted formation of magnetic order in these chains, the existence of which is still debated. Here we report an experimental study of the magneto-conductance (MC) and anisotropic MC with atomic-size contacts and mono-atomic chains of the nonmagnetic metal platinum. We find a pronounced and diverse MC behaviour, the amplitude and functional dependence change when stretching the contact by subatomic distances. These findings can be interpreted as a signature of local magnetic order in the chain, which may be of particular importance for the application of atomic-sized contacts in spintronic devices of the smallest possible size.

## Abstract (web summary)

The development of magnetism in metallic atomic chains is a widely debated phenomenon, of relevance to atomic-scale spintronics. Here, Strigl *et al*. measure the magneto-conductance of platinum break junctions stretched over sub-atomic distances, evidencing the evolution of distinct magnetic order.

## Graphical abstract

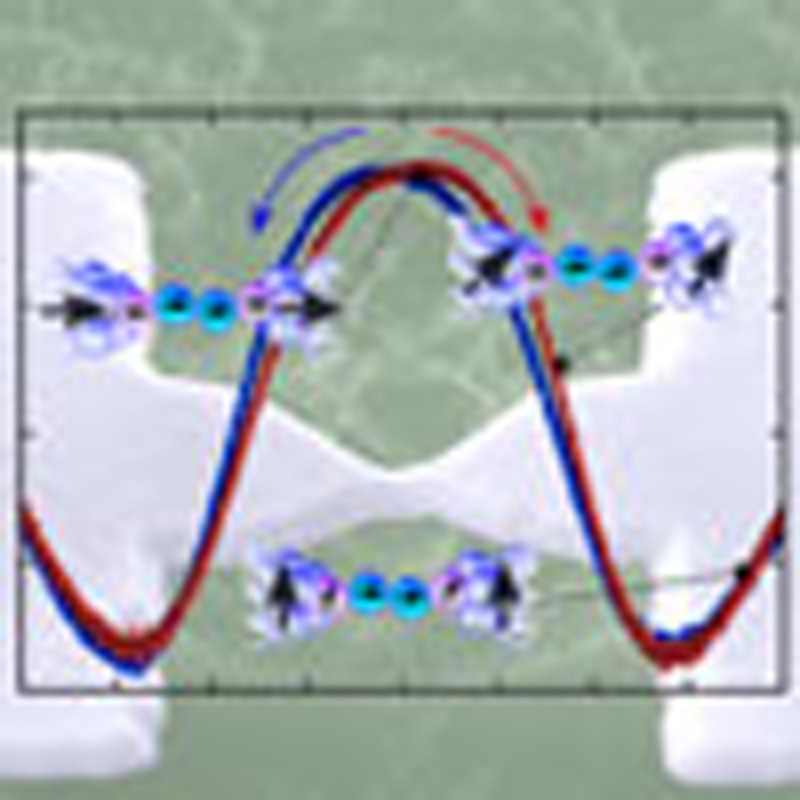

Bulk platinum shows an enhanced magnetic susceptibility, indicating proximity to ferromagnetism according to the Stoner criterion^1^. Upon reduction of the size down to the nanometre scale, this proximity is enhanced and eventually overcomes the Stoner criterion^2^, rendering Pt ferromagnetic at the nanoscale. First experimental support for this emerging magnetism at reduced dimensions arose from work on Pt clusters, where large magnetic moments were identified that increase inversely proportional to cluster size^3^,^4^. A characteristic property of Pt is the formation of mono-atomic chains^5^,^6^, when pulling apart a thin Pt wire, as indicated by the appearance of long plateaus in the stretching curves at a conductance corresponding to the single-atom contact^7^. These mono-atomic chains have been thoroughly investigated theoretically as truly one-dimensional model system. It has been predicted^8^,^9^,^10^,^11^ that the ground state of an atomic Pt chain is ferromagnetic. Experimentally, however, the existence of local magnetic order in atomic contacts is difficult to measure directly, because the sole occurrence of conductance values at half-integers of the conductance quantum *G*_0_ is no proof of spin-polarization or magnetic order^12^. The same limitation holds for the observation of an enhanced monotonic and hysteretic orbital magneto-conductance (MC) in contacts with cross-sections of several nanometres of the nonmagnetic transition metal Pd^13^.

In recent experiments, the shot noise^5^ of Pt atomic chains was studied and no indications of a magnetic ground state were found^14^. A detailed theoretical analysis revealed that this does not exclude the existence of local magnetic order, but the electronic states carrying the magnetic information do not contribute to the shot noise^15^.

For atomic-size contacts of 3d ferromagnets, very large MC and anisotropic MC (AMC)^16^ were observed and attributed to spin-dependent scattering^12^,^17^,^18^,^19^,^20^. However, different origins of the large MC in magnetic nanocontacts have been proposed, such as instabilities caused by magnetostriction^21^,^22^ or quantum interference effects^23^. Nevertheless, a pronounced non-monotonic AMC always requires the existence of a spontaneous magnetization^24^ and MC effects caused purely by orbital motion of electrons in non-magnetic metals are much smaller and always monotonic^16^. Atomic Pt chains represent a unique system, where locally ordered, magnetic moments in the chain are coupled directly to paramagnetic leads. This enables a direct study of the magnetic and transport properties of an atomic-size magnet, the properties of which are not induced or dominated by the magnetism in bulky leads.

Here we investigate the magnetotransport of atomic Pt contacts and chains at low temperature. We observe pronounced features in the MC and AMC curves that are subject to subtle changes of the atomic configurations. The analysis reveals the formation of local magnetic moments in the constriction, and therefore indicates a local origin of the observed magnetotransport effects. Our findings thus support so far unconfirmed theoretical predictions. Furthermore, they show a route to create novel atomic-scale electronic devices based on the direct vicinity of magnetic and nonmagnetic subsystems.

## Results

### Fabrication of single-atom contacts and atomic chains

**Figure 1:**
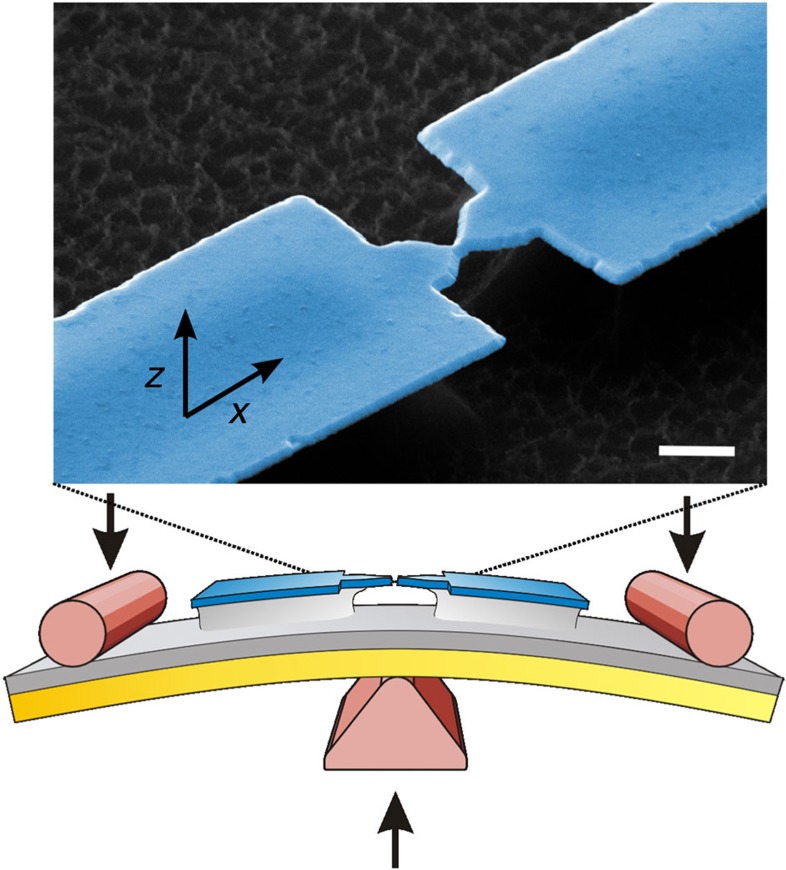
Formation of mono-atomic chains. Mono-atomic Pt chains are prepared using the mechanically controllable break-junction technique, where a suspended, microfabricated metal bridge is carefully bent to adjust the electrode separation. The inset shows an electron micrograph (scale bar, 1 μm) of a Pt junction before opening the contact with a typical cross-section of the constriction of 150 nm. The axes *x* and *z* indicate the in-plane and perpendicular directions, under which the external magnetic fields *B*_*x*_ and *B*_*z*_ are applied.

Here, we investigate atomic contacts by stretching free-standing bridges of Pt fabricated via the mechanically controlled break-junction (MCBJ) technique^25^. A schematic view of the experimental setup and an electron micrograph of a suspended Pt nano-bridge are shown in Fig. 1. Details on the sample preparation and measurement procedures are described in the Methods section. The conductance of a pristine Pt nano-bridge before breaking is typically around 200 *G*_0_, where *G*_0_ denotes the conductance quantum *G*_0_=2*e*^2^*h*^*−1*^ with the elementary charge *e* and Planck’s constant *h*. We adjust the cross-section of the junction by carefully stretching the Pt bridge while measuring its conductance. A single atomic contact is created, when the conductance drops below 2.5 *G*_0_ (ref. 7); further stretching of the contact results in mono-atomic Pt chains. Using this technique, Pt chains of up to seven atoms in length can be created repeatedly^5^,^7^. The MC of these contacts and chains is measured at a constant length, sweeping the magnetic field parallel (*x* direction) or perpendicular (*z* direction) to the direction of the electrical current. We observe five characteristic features that indicate magnetic order, which are discussed below in separate paragraphs and compared with reference measurements on gold break junctions, where no magnetic ordering occurs in reduced dimensions.

### Emergence of manifold shapes in MC of few-atom contacts

**Figure 2:**
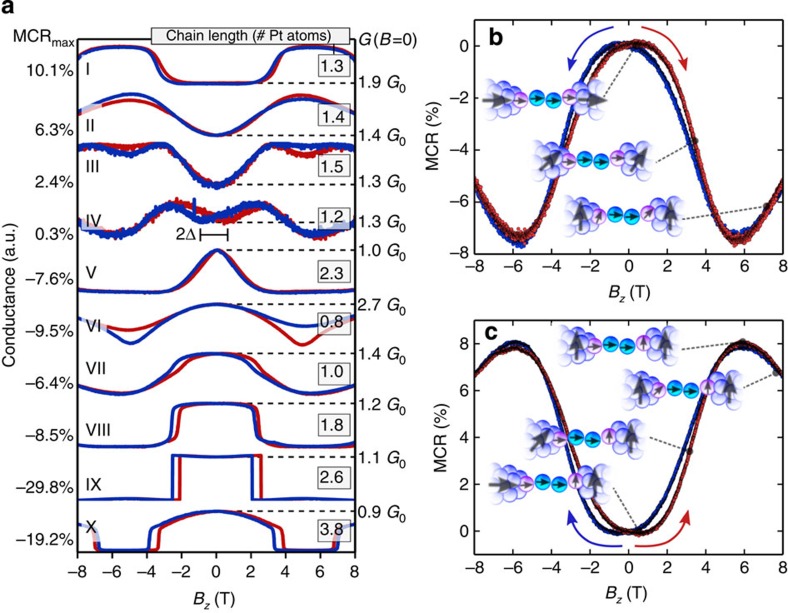
Magneto-conductance traces of atomic Pt contacts. (**a**) Collection of magneto-conductance (MC) traces recorded in single-atom Pt contacts and chains. The curves are shifted relative to each other and scaled to similar amplitudes for better visibility. The magnetic field *B*_*z*_ is applied perpendicular to the sample plane up to a maximum value of ±8 T. The magnetic field is swept from −8 T to 8 T (red curves) and back to −8 T (blue curves). We observe a large diversity in shape, sign, amplitude and hysteresis of the MC traces; for comparison, the maximum magneto-conductance MCR_max_, the chain length in units of atomic diameters (boxed and superimposed close to the right axis) as well as the zero-field conductance (dashed lines and labels to the right of the panel) are indicated for each curve. The hysteresis (2Δ) is defined as the separation of the low-field extremes as indicated for curve IV. **b** and **c** exemplify the most common MC curves and illustrate a simple model to qualitatively understand them on the basis of different relative alignments of magnetic moments; the insets depict schematically the orientation of moments in the constriction. The outermost chain atoms connecting directly to the leads (blue) are referred to as apex atoms (purple spheres), the other chain atoms are shown as cyan coloured spheres. The arrows indicate a possible orientation of magnetic moments in the constriction. In **b**, the state at low field is a magnetization of both the central chain and the apex atoms along the chain axis (*x*-direction). In **c**, the low-field state is a magnetization of the central chain and one apex atom along the wire, the other apex atom perpendicular to the chain and the electrodes follow the magnetization of the adjacent apex atom, which leads to a rather low-conductance state.

First, we record MC traces in magnetic fields up to |*B*_*z*_*|*<8T perpendicular to the sample at 4.2 K. We concentrate on Pt single-atom contacts or chains with *G*~0.9 to 2.5 *G*_0_. For large contacts with *G*>30 *G*_0_, the MC shows a weak, negative field dependence, as expected for bulk Pt^16^ and as shown in Supplementary Fig. 1. For small contacts with cross-sections of few atoms (*G*<5 *G*_0_), we observe MC curves similar to those found in atomic-size contacts of 3d band ferromagnets^12^,^17^,^18^,^19^,^20^. Typical examples of such MC traces are collected in Fig. 2a. The occurrence of MC curves with different shapes and magnitudes serves as a first indication for local magnetic order in Pt chains. To quantify the MC effects, we define the MC ratio (MCR) as the conductance variation relative to the conductance close to zero field: MCR(*B*)=(*G*(*B*)−*G*_ext_(*B*≈0))/*G*_ext_(*B*≈0), where *G*_ext_(*B*≈0) is the extreme value of conductance at low field. Owing to hysteresis effects, which are discussed below, the reference value *G*_ext_(*B*≈0) is not necessarily at zero field. The MCR_max_ values indicated in Fig. 2a are defined as the largest deviation in the MC trace from *G*_ext_*(B*≈0) normalized to *G*_ext_*(B*≈0). In Fig. 2a, the stretching length at which the curves have been recorded as well as the zero-field conductance are indicated for each curve. All curves show MC changes occurring on a field scale of several Tesla, all are non-monotonic and hysteretic. In the curves I–III and VI additionally the amplitudes of the finite-field extremes depend on the sweep-direction, which is typically observed in magnetic tunnel junctions (MTJs) and is attributed to a spin-polarization of the electrodes^16^. However, the field scales are typically much smaller for MTJs of band magnets. Figure 2b,c shows examples of the two most frequently observed MC traces (see Supplementary Table 1 for details about the relative abundance of the shapes). In one case (Fig. 2c), we observe a positive MC with a minimum conductance near zero field, whereas in the other case (Fig. 2b), the MC is negative with a maximum conductance close to zero. The insets depict a simple model we develop herein, which indicates how an external magnetic field may influence the local magnetic moments of a contact, leading to the observed MC. This model is described in detail in the Discussion section.

### Sensitivity of MC to atomic configuration

**Figure 3:**
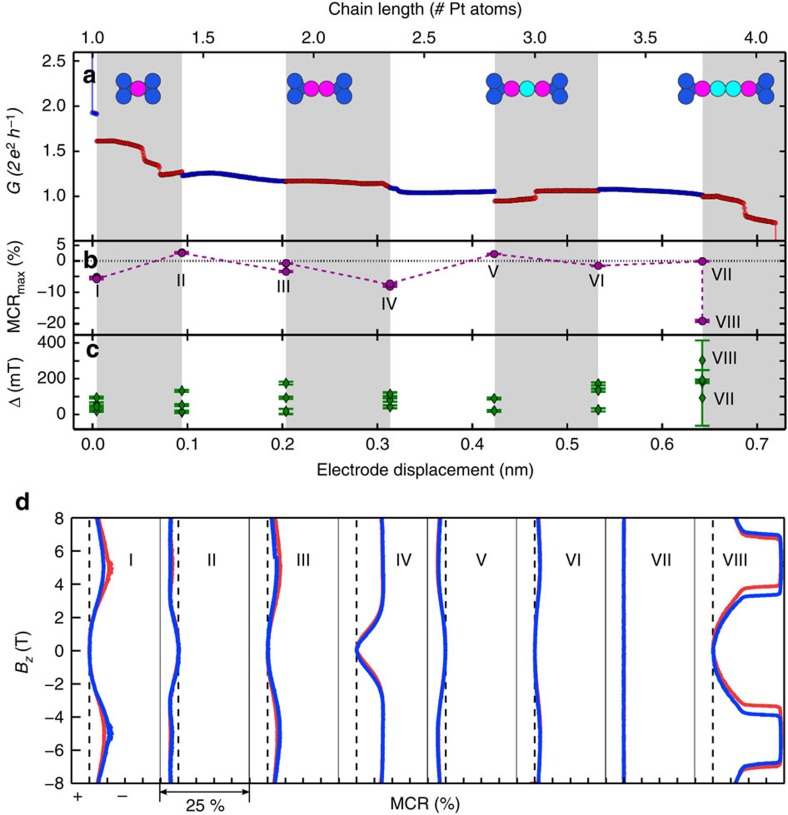
Development of the magneto-conductance during evolution of a Pt chain. (**a**) Stretching curve of a Pt bridge interrupted at regular intervals to measure the magneto-conductance (MC). The sample was opened continuously until the single-atom contact regime at conductance *G*<2.5 *G*_0_ was reached. Then several MC traces in perpendicular (*B*_*z*_) fields up to ±8 T were recorded at fixed chain length, while stepwise opening the contact at intervals of approximately 2/5 of a Pt atomic diameter in a chain as indicated by the shaded regions. The insets sketch the atomic chain (cyan spheres) coupled to the electrodes (blue) via apex atoms (purple) as a number of Pt atoms equivalent to the electrode assuming a rigid chain with a bond-length of 2.33 Å. **b** shows the maximum MC ratio (MCR_max_), and the hysteresis (Δ) is shown in **c**; these values are extracted from individual MC traces exemplified in **d**. The symbols in MCR_max_ and Δ represent consecutive measurements at fixed opening positions. MCR_max_ varies between values close to zero and up to 20% as the chain is created. Moreover, the MC continuously changes its sign depending on the atomic configuration of the contact. The hysteresis also changes non-monotonically with the chain length between 20 and 300 mT. (**d**) Out-of plane MC traces for different contacts recorded along the stretching curve at positions I–VIII used to extract the MCR_max_ and Δ values shown in **a**. The horizontal axis spans the range from +5% to −20% in all panels, the dashed line indicates zero magnetoresistance amplitude (MCR).

Next we follow the development of the MC traces while stretching a mono-atomic chain, see Fig. 3. When arriving at the single-atom contact regime (*G*<2.5 *G*_0_), we interrupt the stretching at intervals of about 0.11 nm, which corresponds to 38% of an atomic diameter, to record series of MC curves at each fixed chain length. Figure 3 shows the evolution of the conductance in zero field (Fig. 3a) as well as the amplitude MCR_max_ (Fig. 3b) and hysteresis Δ (Fig. 3c) of the MC curves; the stretching intervals are highlighted as alternating shaded regions. The multiple symbols for MCR_max_ and Δ at a fixed chain length in Fig. 3b and c correspond to subsequent magnetic field sweeps, in which we observe only a small variation of the curve shape, hysteresis and MCR_max_. The MCR-shapes corresponding to the MCR_max_-values labelled with roman numerals (I–VIII) in Fig. 3b are shown in Fig. 3d. The MC traces VII and VIII in Fig. 3d were recorded at the same nominal chain length of 3.8 atoms but both are drastically different. We observed such a large difference in subsequent MC measurements in particular for ultimately stretched Pt chains and attribute this behaviour to a lower stability and a larger sensitivity of these chains to magnetic field-induced variations of the contact geometry. Owing to this reduced stability the contact breaks while extending the nominal chain length above 4.1 atoms.

While extending the length of mono-atomic chains starting from the single-atom contact, the sign, amplitude MCR_max_ and hysteresis Δ as well as the shape of the MC traces (Fig. 3d) change within subatomic variations of the chain length, whereas the overall conductance varies only slightly. Even a small variation of the electrode separation can induce changes in the atomic contact configuration. However, in single-atom contacts and chains, the conductance is rather independent of length. Hence, when an additional atom is extracted from the electrodes to join the mono-atomic chain, the bond-length between the chain atoms and the electrodes change, whereas the absolute conductance remains fairly constant. However, the magnitude, anisotropy direction and coupling of magnetic moments in the chain are very sensitive to the bond length^8^,^26^,^27^. The observed variation of sign and shape of the MC curves upon stretching the contact by fractions of an atomic diameter hallmarks this high sensitivity. In the example shown in Fig. 3b, the MCR_max_ values vary between 2 and −20%. For other chains, similar results and MCR_max_ up to ±40% were found (see Supplementary Fig. 2). Both the large amplitude of the MC and its sensitivity to sub-atomic length changes point towards a local origin of the MC rather than a bulk effect and serve as a second indication for magnetically ordered Pt chains.

### Hysteresis of magnetoconductance curves

**Figure 4:**
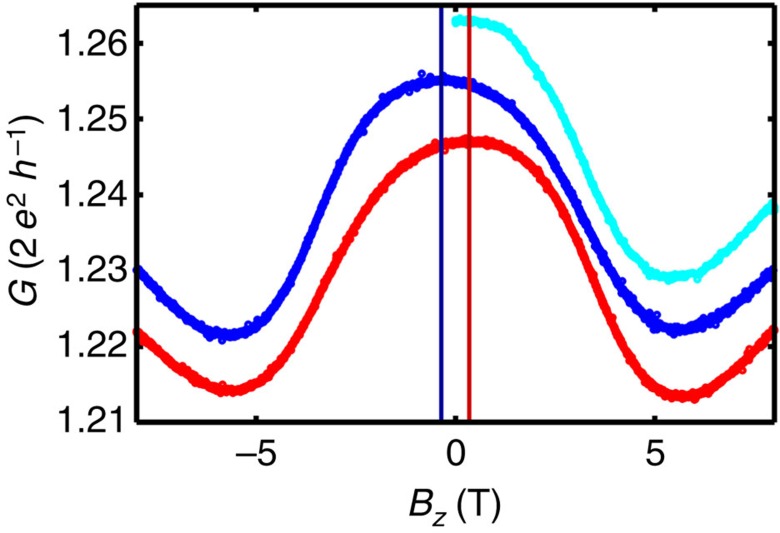
Emerging hysteresis in an atomic Pt contact. After adjusting the contact the MC shows an extreme at zero while ramping the magnetic field the first time (cyan curve). After application of a high field, this minimum is shifted by about 350 mT in sweep direction when performing a magnetic field sweep. Please note that the curves have been shifted vertically for a better visibility. The cyan curve has been measured right after adjusting the contact when sweeping the field from 0 to+8 T, then the blue curves when sweeping from +8 T to −8 T and finally the red curve when sweeping from −8 T up to +8 T.

Most MC traces recorded for Pt single-atom contacts and chains reveal a hysteretic behaviour of the conductance with a local extreme *G*_ext_*(B*≈0) located symmetrically around zero field, as indicated by the red and blue curves in Fig. 4. Here we define the hysteresis Δ as the deviation of position of this local extreme from zero field, as indicated by the vertical markers in Fig. 4. The numerical values of the hysteresis are calculated by fitting a quadratic polynomial to the area around the central extreme *G*_ext_*(B*≈0), the vertex of the polynomial marks the hysteresis, whereas the standard deviation of the best-fit result represents the error bars shown in Fig. 3c. When applying a magnetic field starting from *B*=0 on a freshly adjusted contact, that is, after rearranging the contact geometry, we mostly find an extreme value *G*_ext_*(B*≈0) of the conductance exactly at zero field, see cyan trace in Fig. 4. The position of this extreme value is shifted along the field axis by a fixed value, which depends on the contact, during consecutive magnetic field sweeps with high amplitude. The size of this hysteresis Δ, ranges between zero and 800 mT, and is independent of the sweep rate, but varies from contact to contact, even while stretching a chain in subatomic intervals, where the status of the leads is not affected, see Fig. 3c. However, the contact-to-contact variation of the hysteresis within one chain is small (<300 mT), see Supplementary Fig. 2, whereas the hysteresis variations from chain to chain are more significant. The size range of the hysteresis is different for all chains and seems to depend on the opening history, whereas its exact value depends on the configuration of the single-atom contact. The strong dependence of the hysteresis on the atomic arrangement of the constricted electrodes, which is defined during the formation of the single-atom contact and changes only subtly when extending the chain, suggests that the hysteresis is attributed to the anisotropy energy of the electrodes. Hence, a large variation of Δ from chain to chain is expected because the junction is closed completely before adjusting a new single-atom contact and therefore the contact geometry changes completely. In the control experiments with gold atomic chains (see below), we did not find such behaviour. Therefore, we interpret the hysteresis in Pt chains as a measure for the coercive field of the magnetized electrodes in the vicinity of the contact and hence the development of a local magnetically ordered state in Pt chains. This interpretation is also supported by the markedly different MC in *x*-direction, which strongly depends on the history of the chain, that is, on the application of a large magnetic field in *z*-direction. This anisotropy of the MC is discussed in the following section.

### Functional shape of AMC curves

**Figure 5:**
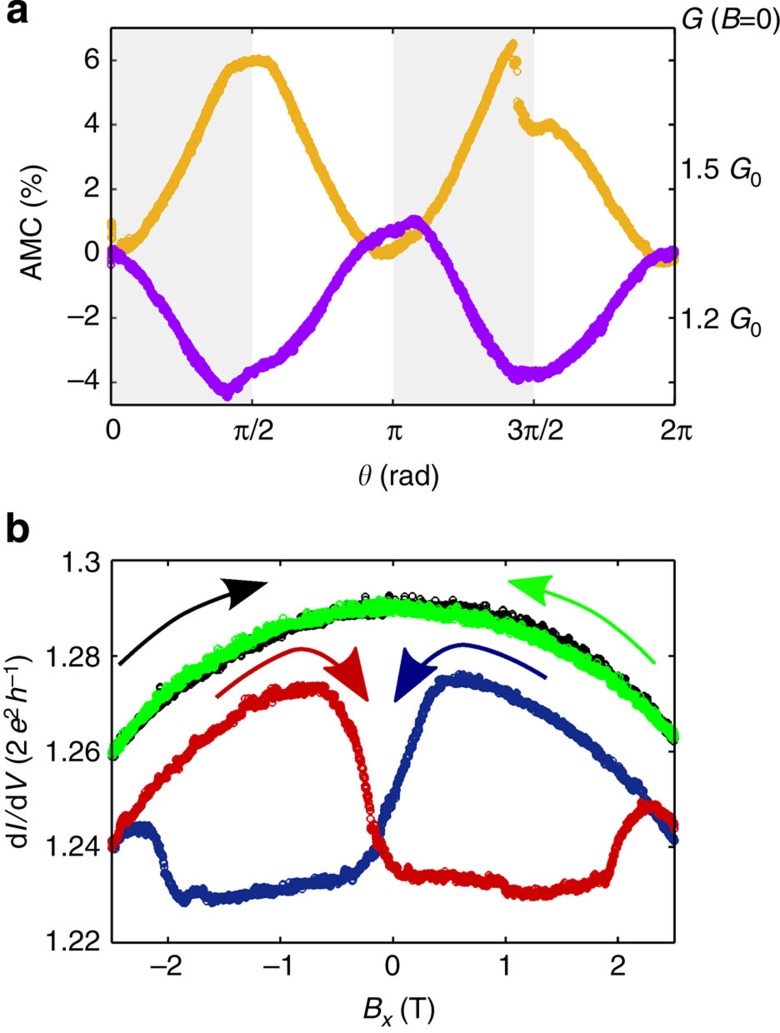
Anisotropic magneto-conductance and in-plane magneto-conductance of Pt chains. (**a**) The magneto-conductance (MC) as a function of the angle (*θ*) between current direction and magnetic field applied in the *xz*-plane shows an anisotropic behaviour for different contacts. *θ*=0 corresponds to a magnetic field in the *x* direction. The anisotropic magneto-conductance (AMC) traces were recorded for two contacts at 1.52 *G*_0_ (orange) and 1.23 *G*_0_ (purple) during chain stretching at an applied field of 2.5 T. The signature approximately follows a ±cos^2^*θ*-shape. (**b**) Atomic Pt contacts display a history-dependent effect of the MC. The red and blue curves show the in-plane (*x*-direction) MC of an atomic contact immediately after a magnetic field *B*_*z*_=7 T was applied and then removed again. The arrows indicate the sweep direction. We observe a pronounced MC with amplitudes of up to 6%, which is comparable to the maximum MC ratio (MCR_max_) observed in perpendicular field. However, if no prior *B*_*z*_ was applied or the chain is left at zero field for several hours after a *z*-sweep, we observe only a parabolic dependence of the conductance on the magnetic flux (black and green traces).

The fourth indication supporting a magnetically ordered state is the presence of a strong AMC. In bulk ferromagnets, the AMC is typically only one per cent and even smaller in saturated paramagnets^16^. A strong increase of the AMC in ferromagnets with reduced dimensions has been reported experimentally and theoretically^12^,^19^,^21^,^24^. A key consideration is that the orbital moments of electrons, which are generally quenched in the volume, are not negligible in nano-contacts of atomic dimensions, because of a stronger spin-orbit scattering^16^,^18^. We study the AMC of Pt contacts by rotating the direction of the applied magnetic field (2.5 T) in the *xz*-plane over a 2π angle, see Fig. 5a. The AMC shows the usual cos^2^*θ*-dependence with large amplitudes of 4–6%. While stretching the chain, the general shape of the AMC remains unaffected but its sign changes. This behaviour is incompatible with the bulk theory of AMC^16^, but can be explained by spatial asymmetries and by spin polarization of the conduction channels^24^. This model put forward for atomic contacts of band ferromagnets predicts a manifold AMC behaviour, including sign changes due to atomic reconfiguration and large amplitudes that are similar to our experimental findings. Therefore, the large AMC and the observed sign changes strongly imply that the conduction channels in Pt contacts are indeed spin-polarized by the emergence of a local magnetically ordered state.

### Dependence of MC on magnetic history

The fifth indication for a local magnetic order is the observation of history- and time-dependent in-plane MC (*x*-direction). We observe a symmetric, parabolic MC (green and black traces in Fig. 5b), if only a moderate |*B*_***z***_|<2.5 T field is applied before measuring the in-plane MC, where the magnetic field is applied parallel to the current. However, if a strong, perpendicular field of |*B*_***z***_|>7 T was applied before recording the in-plane MC, a highly asymmetric and strongly hysteretic behaviour is observed (blue and red traces in Fig. 5b). The extreme values of the up- and down-ward sweeps occur at fields, which are in the same order of magnitude as the hysteresis observed in the out-of-plane MC traces, suggesting that the in-plane MC is governed by the magnetic properties of the electrodes. The MC relaxes, at the low temperature of our measurement over time scales of several hours, pointing again towards a local magnetic order^16^ rather than a bulk effect. The relaxation of the MC over such long time scales is interpreted as a relaxation of the perturbed magnetization state after the application of a large out-of-plane field to its ground state, where the magnetic moments are preferably aligned in-plane.

### Reference measurements on gold atomic contacts

**Figure 6:**
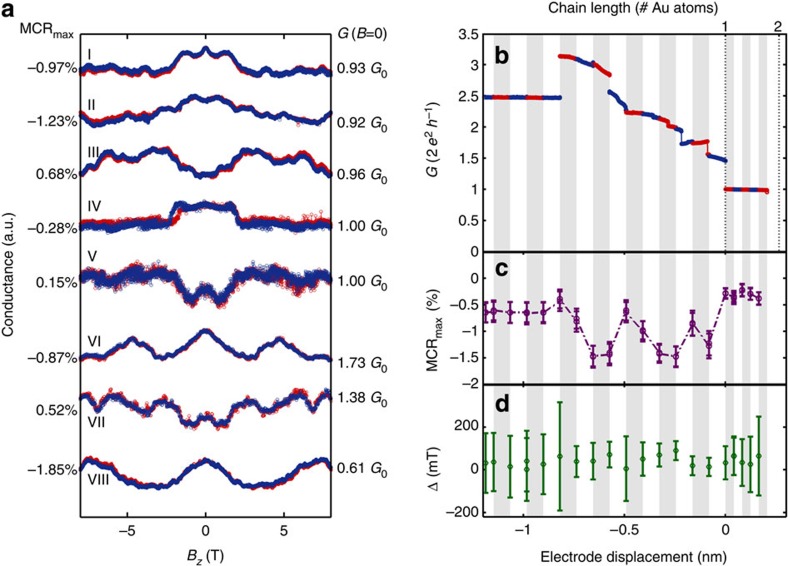
Magneto-conductance traces of atomic Au contacts and the development of the magneto-conductance during evolution of a Au chain. (**a**) A collection of magneto-conductance (MC) traces of a Au sample. The traces I–V show contacts with a conductance close to 1 *G*_0_, traces VI–VIII contacts with non-integer conductance. For contacts around 1 *G*_0_, the maximum MC ratio (MCR_max_) scarcely exceeds 1%, whereas for contacts of non-integer conductance, slightly higher amplitudes occur. The overall effects are considerably smaller than for Pt, and can be attributed to quantum interference effects (universal conductance fluctuations). The single traces have been normalized and shifted vertically for clarity. (**b**) The evolution of the MC while opening an Au contact starting from a few-atom contact with 2.5 *G*_0_ down to a single-atomic contact around 1 *G*_0_ and further until it breaks. The stretching of the contact was stopped in regular steps and magnetic field sweeps to ±8 T were performed at each step. The MCR_max_ and hysteresis of each contact are displayed in **c** and **d**. The MCR_max_ exceeds 1% solely where the conductance changes significantly during stretching, but becomes considerably small when the conductance drops to ~1 *G*_0_, where a single-atomic contact is formed. The hysteresis Δ ranges between 20 and 80 mT, but since the MC ratio (MCR) signal is small, the errors are very large.

The general MC behaviour of Au differs drastically from those of Pt, although both metals form mono-atomic chains when stretching a single-atom contact. A collection of MC traces for Au single-atom contacts and chains is shown in Fig. 6a. Most traces reveal small MCR_max_<1% and changes of MC on much smaller field scales, which are reminiscent of conductance fluctuations^28^ in atomic contacts. The amplitude is not expected to show the universal value because of the non-homogenous voltage drop^29^,^30^,^31^. Similar to Pt, we find a large diversity of MC shapes and amplitudes, which occasionally change sign. Overall, there is a clear tendency for negative MCR_max_. Nevertheless, similar shapes than in Pt chains can be identified, but the amplitude of the MC effect is approximately one order of magnitude smaller. For example, the shape of trace III in Fig. 6a shows similarities to trace III in Fig. 2a. Despite the occurrence of similar shapes of MC traces in Pt and Au atomic contacts and chains, in the case of Au we did not find a distinct curve shape that is more prominent than others. This is in contrast to Pt, where the two traces shown in Fig. 2b,c represent the most abundant form of MC traces observed in these contacts. In Au contacts and chains with a total conductance near 1 *G*_0_, the MCR_max_ values hardly exceed 1%. This changes when the zero-field conductance differs markedly from multiples of *G*_0_, that is, when partially opened conduction channels contribute, or when the conductance drops below 1 G_0_. For a contact of 0.6 *G*_0_, we found a MCR_max_ value of more than 1.85%, see Fig. 6a trace VIII. These findings are in agreement with earlier reports of conductance fluctuations of Au atomic contacts and changes studied upon sweeping the bias voltage^32^.

As for Pt, we study the evolution of the MC and its hysteresis while extending a Au chain, starting from the single-atom contact. Again, the chain length is adjusted and then kept constant, while a series of MC traces is recorded before stretching the chain further. Figure 6 shows an example of the development of the zero-field conductance (Fig. 6b), the MCR_max_ (Fig. 6c) and the hysteresis Δ (Fig. 6d) of a Au chain. Like for Pt chains, occasionally we observe an instantaneous switching behaviour on Au chains as well, which can be interpreted as arising from atomic rearrangements, but leading to a comparably small MC of 2.6% compared with 30% on Pt, see trace IX in Fig. 2a. To determine the hysteresis, the centre parts of the sweeps are evaluated in the same manner as for Pt. However, generally the MC traces of Au chains are only weakly or non-hysteretic. The exact evaluation shows Δ in the range of 20 mT, which occasionally increases up to 80 mT, however, the error bars are much larger than in the case of Pt. In summary, the MCR in Au is extremely small and non-hysteretic, while universal conductance fluctuations account for most of the conductance variation observed in these contacts. A larger set of opening and MC traces for Au is shown in the Supplementary Fig. 3. The fact that no clear MC is observed for Au samples proves that the large MC observed in Pt contacts is no artefact of the measurement setup and supports the argument that in Pt mono-atomic chains a local, magnetically ordered state develops.

## Discussion

Although the experimental observation presented above clearly indicate the formation of magnetic order in atomic Pt contacts and chains, the exact interpretation of the shapes of the MC curves remains a difficult task. We propose a qualitative understanding: following the results of theoretical calculations for atomic contacts of band ferromagnets, we assume that a parallel alignment of the magnetization gives rise to the highest conductance, whereas a misalignment reduces the conductance^33^. This approach is similar to Jullière’s model for MTJs^16^. In contrast to MTJs, where the field scales are set by the coercivity of the electrodes separated by a nonmagnetic barrier, in Pt atomic contacts the magnetism, if present, is created locally on atomic length scales. Different magnetization directions of mono-atomic Pt chains have been predicted with magnetic moments per atom ranging from 0.2 to several *μ*_B_ (refs 8, 9, 10, 26, 27, 34, 35). Most calculations agree that the magnetization in the ground state is parallel to the chain axis (insets Fig. 2b), but for certain bond lengths, which can be realized by stretching a chain, the magnetization may become perpendicular to the chain axis^26^,^27^,^35^. Very high values of more than 100 meV were predicted for the magnetic anisotropy energies (MAEs) of the chain and apex atoms, but with varying magnetization directions^26^. Within these calculations, the energetic ground states of the individual atoms for different bonding lengths in the chain were determined separately, before including their interaction. Although the magnetic moment of the central atom is generally pointing in chain direction, those of the apex atoms preferentially lie perpendicular to it. Finally, the coupling among these atoms and the coupling to the electrodes lead to a reduced MAE in the range of several tens of meV for the entire chain, which corresponds to a Zeeman field of >30 T (refs 26, 35). As a result, for small atomic distances, the competing energy scales are of comparable size and might thus lead to a non-collinear magnetization in the ground state (insets Fig. 2c)^26^,^36^. Although such a ground state has not been considered explicitly for pure Pt samples^26^, the mechanism of strong spin-orbit coupling of Pt leading to a spin torque on the magnetic moments of small clusters and chains of 3d band magnets^36^ should be the same for magnetic moments in atomic Pt chains. The structure of magnetic moments in atomic Pt contacts might be even more complex because the calculations did not take into account the influence of the conical leads^9^,^27^,^35^, which have been shown to carry a finite magnetic moment^37^ that is weakly pinned but affects the magnetization of the apex atoms. Furthermore, we assume that the magnetic moments of the chain, apex and central atoms polarize the innermost parts of the electrodes within a range of several atomic layers^26^,^38^. Following these considerations, the total MAE is reduced and moderate magnetic fields <10 T should be sufficient to reorient the moments of the electrodes and the apex atoms, while the central chain atoms remain strongly pinned along the chain direction and would not be affected by the magnetic fields accessible experimentally.

Acknowledging the possibility of non-collinear magnetization, we depict a possible scenario which qualitatively describes the MC curve of Fig. 2b: in the ground state, all magnetic moments are aligned along the chain axis. Applying an external field perpendicular to the chain axis results in a relative misalignment between electrodes and chain at field scales corresponding to the anisotropy energy of the electrodes, roughly 400 mT in this case, and thus to negative MC. The conductance change occurs gradually rather than abruptly as in MTJs because several energy scales including the anisotropy energies of the components of the device and the coupling between them are involved. When the magnetic field is increased further to overcome the anisotropy energy of the apex atom (here: 5.5 T), their moments are pulled out of the chain axis and depending on the combined contribution of AMC and conductance change due to misalignment, the conductance may decrease further, remain constant (as, for example, observed in curve V of Fig. 2a) or increase as in Fig. 2b. In the case of a non-collinear ground state, for example, if, at zero field, the chain atoms, one electrode and one apex atom are magnetized in plane, while the other apex atom and the adjacent electrode are magnetized out of plane (inset Fig. 2c), the application of a moderate field initially results in a positive MC, until the electrodes are aligned parallel with respect to each other. At large fields >6 T, the moment of the in-plane apex atom may reorient out of plane and due to the misalignment with the moments of the central chain atoms, the conductance decreases again. The sweep-direction-dependent conductance values (curves I–III and VI in Fig. 2a) can be explained assuming a non-collinear arrangement of the apex atoms in the ground state. The appearance of additional extremes as exemplified by traces III and IV may be understood by the subtle interplay between the MAEs of apex, chain and electrode moments. Using this simple model, based on the reorientation of magnetic moments in the chain at different field scales, an interpretation of all MC curves in Fig. 2a is presented in Supplementary Fig. 4 and the Supplementary Discussion. In conclusion, our comprehensive set of observations in atomic Pt chains clearly indicates the formation of local magnetic moments in the constriction. The magnetic field influences their coupling and thereby also the conduction channels resulting in pronounced features in the magnetotransport characteristics that are subject to subtle changes of the atomic configurations. Our findings do not only confirm the longstanding theoretical predictions of local magnetism in atomic chains, but they also reveal why subtle differences in the theoretical model may lead to different predictions for the magnetic order and give valuable input for the still lacking calculations of magnetotransport in these structures. The proposed interpretation model and the occurrence of diverse MC shapes imply that a non-collinear alignment of magnetic moments in atomic Pt contacts is a very common feature. The possibility of creating local magnetic order in ultimate proximity to nonmagnetic electrodes is particularly interesting for the development of atomic-scale magnetoelectronic and spintronic devices, because spin-polarized currents can be created and detected on the atomic scale in ultimate vicinity to nonmagnetic devices.

## Methods

### MCBJ technique

The cross-section of the junctions required for the experiments described herein must be continuously tuneable *in-situ*. The samples need to provide sufficient long-time stability to hold a single-atomic contact over periods up to several days. The first requirement demands for high flexibility, whereas the latter one is mandatory to perform magnetotransport measurements without changing the geometry by mechanical excitation, heating, magnetostriction or other effects. We chose the method of lithographically defined MCBJ because they present a good compromise between tuneability and stability. In principle, also STM-based junctions are suitable candidates with the benefit that information about the atomic arrangements of the surroundings can be determined. However, the low stability of the STM setup, in particular when applying external magnetic fields in various directions, limits this approach to relatively short-term studies of large numbers of contacts. In contrast, the MCBJ technique has the advantage that single-atom contacts can be stabilized over extended periods of time without large perturbation from external factors, but the high stability also means that statistical measurements become very time consuming.

### Standard lift-off process for Au

A layer of polyimide (2 μm thick) is spin-coated onto a polished bronze wafer (200 μm in thickness). This layer serves as an electrical insulator and a sacrificial layer in the subsequent etching process. On top of the polyimide, a double layer of electron-beam resists, methyl methacrylate-methacrylic acid (MMA-MAA)/polymethyl methacrylate (PMMA), is deposited by spin-coating (maximum 5,000 r.p.m.). Then electron-beam lithography (EBL) is performed on the prepared wafer. After developing the resist, a layer of Au is deposited with a thickness of 80 nm via electron-beam evaporation at a pressure of 10^*−*8^ mbar. Redundant parts of the metal film are removed by lift-off in acetone at 50 °C. To finalize the samples, they are dry etched in oxygen plasma of 50 W in a reactive ion etcher, which results in a free-standing bridge^39^.

### Subtractive patterning process for Pt

The fabrication of Pt samples by using the lift-off process is not very reproducible. Because of the high boiling point of Pt, the PMMA is modified during evaporation, causing the lithographically defined constriction to collapse. Cooling the sample with liquid nitrogen during evaporation process did not result in improved quality of the samples. The main reason is that owing to the different thermal expansions of the polyimide and the metal film, the constriction breaks while warming up the sample after evaporation. To overcome this problem, we use subtractive patterning via dry etching^25^. A pristine Pt layer (80 nm) is deposited before the EBL process on a polyimide layer via sputter deposition (base pressure ~10^*−*5^ mbar). The double-layer electron-beam resist (MMA-MAA/PMMA) is deposited on the Pt film in order to perform the EBL. After developing the resist, an Al layer with a thickness of 30–35 nm is deposited via thermal evaporation at a pressure of 10^*−*8^ mbar. After lift-off in acetone at 50 °C, the samples are placed in a reactive ion etcher, where a highly anisotropic etching process at 150 W with sulphur hexafluoride (SF_6_) as a reactive gas is performed to remove the parts of the Pt film that are not protected by Al. Similar to the standard process, a free-standing bridge is formed via isotropic etching with oxygen plasma. The sample is placed in potassium hydroxide solution (KOH) for few seconds to remove the residual Al and then dried with N_2_ gas.

### Electromigration treatment

After cool-down, before breaking the first time, we perform a soft electromigration (EM) process to remove impurities and heal out structural defects in the constriction of the Pt samples. Without EM, the opening traces show very shallow and inclined steps, often at values below 1 *G*_0_, no pronounced jump to tunnelling, and the resulting histograms reveal no or broad peaks reminiscent of the findings for PtC0 (ref. 40). We attribute these effects to contamination of the Pt film during sputtering and the subsequent patterning process. To obtain pure, defect-free Pt junctions, the EM is carried out in cryogenic vacuum and works as follows: An increasing dc voltage of several volts is applied to the sample until a threshold resistance change of 1–10% above the initial resistance is reached. The voltage is then reduced, so that the power dissipated at the sample is approximately 10–70% of the maximum value. This defines the initial condition for the next cycle and voltage is increased again until the threshold of resistance is reached again. This procedure is repeated until a total conductance decrease of a 5–8% is achieved, corresponding to a resistance change of several Ω (ref. 41). An example is given in Supplementary Fig. 5.

One can identify several regimes in the EM process defined by threshold and power reduction. If the threshold is low or the power is reduced to small values, the constriction simply heats up and cools down on temperature scale too low to trigger structural changes. If the threshold is high or the power is reduced only slightly, the sample heats up very quickly and the process becomes unstable, which in many cases leads to a destroyed sample via melting or evaporation of the central constriction. In the intermediate regime, both the desired healing effect and a controlled thinning of the cross-section of the junction take place. The optimum parameter range varies from sample to sample and may change for a given sample depending on the history of the EM process. We found the healing process to be completed after a resistance change of 5–8% relative to the initial resistance. When continuing to perform the EM, either no further resistance change was observed or an increasing resistance signalling a thinning process without further reduction of impurity concentration^42^,^43^.

### Magnetotransport measurements

The data set comprises measurements of the MC in two different directions of the applied field with respect to the sample plane and measurements of the AMC in a wide range of conductance values from 50 *G*_0_ to 0.001 *G*_0_. The measurements are performed at low temperature (<6 K) while continuously changing the contact geometry on the atomic scale. Besides the adjustable and stable samples described above, this requires simultaneously a very stable and nonmagnetic MCBJ mechanism, very good thermal anchoring to avoid eddy-current heating, as well as very sensitive electronics to measure conductance changes of up to six orders of magnitude. A sketch of the MCBJ mechanism is shown in Supplementary Fig. 6. For all measurements, both dc conductance and differential conductance d*I*/d*V* were measured with a custom-built cryo-electronic setup.

MC measurements were carried out in a ^3^He insert (base temperature 270 mK) with a custom-made mechanically controllable break junction system. Before breaking the junction for the first time, the setup is evacuated to high vacuum and inserted into a helium dewar. The setup features a superconducting solenoid with fields in vertical (*z*) direction of up to 8 T, and a superconducting split-coil magnet in horizontal (*x*) direction delivering field up to 3 T. We perform a gentle EM process, as described above, to heal out defects in the constricted region of the nano-bridge, caused by the thin-film deposition and the subsequent fabrication steps. After this electrical annealing, 2–5 c.c.m. of ^4^He exchange gas is introduced into the sample space to thermally couple the sample to the ^4^He bath. Then a mechanical training by stretching and relaxing the bridge is performed using a purely mechanical breaking mechanism, controlled by a dc motor and a differential screw. A scheme of the experimental setup and an electron micrograph of a suspended Pt nano-bridge are shown in Fig. 1. The conductance histograms of Pt, calculated from several hundred breaking curves, feature a broad peak around 1.4. *G*_0_, which is in agreement with the literature^5^,^7^. This also confirms the purity of the samples in the contact region. Further details on the pre-characterization routines are provided in the Supporting Methods. To measure the MC, we usually ramp the magnetic field from zero to the maximum value and then sweep it twice between the maximum and minimum. This enables separating real magnetic field induced effects from temporal drifts and heating effects, but also helps to discard contact instabilities that reveal themselves as sudden conductance jumps. When recording MC traces, the chain is not necessarily in its magnetic ground state. If so, the first sweep (from 0 to *B*_*z*,max_) should reveal a different shape than the subsequent ones. We restrict our interpretation to those MC traces that show similar behaviour in all sweeps. Examples of a stretching curve and a conductance histogram of Pt are shown in Supplementary Fig. 7.

## Author contributions

F.S., C.E. and M.B. performed the experiments. T.P. and E.S. planned the project and advised the students. All authors discussed the results and prepared the manuscript together.

## Additional information

**How to cite this article**: Strigl, F. *et al*. Emerging magnetic order in platinum atomic contacts and chains. *Nat. Commun.* 6:6172 doi: 10.1038/ncomms7172 (2015).

## Supplementary Material

Supplementary Information Supplementary Figures 1-7, Supplementary Table 1, Supplementary Discussion, Supplementary Methods and Supplementary References
