# Supplementary material for: Emerging magnetic order in platinum atomic contacts and chains
Source: Nat Commun. 2015 Feb 4;6:6172. doi: 10.1038/ncomms7172 (PMC4347049; doi:10.1038/ncomms7172)
Supplement: Supplementary Information — Supplementary Figures 1-7, Supplementary Table 1, Supplementary Discussion, Supplementary Methods and Supplementary References [file ncomms7172-s1.pdf]

Supplementary Figures

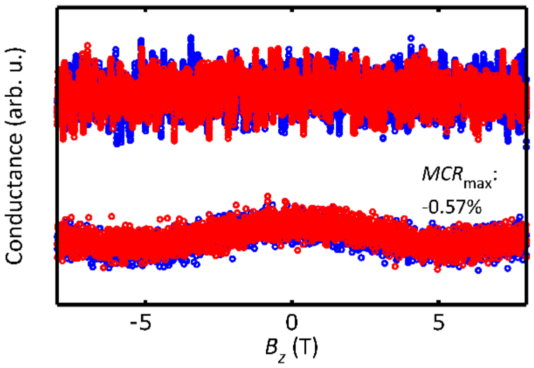

Supplementary Figure 1 | Magneto-conductance traces of a platinum sample while opening the contact. While there is no detectable signal at 130 G_0 (upper trace) a small signal can be found for a contact of 26 G_0 (lower trace). The electronic setup is optimized for measurements in the atomic contact regime and has higher voltage noise for high conductance values. The colours red and blue indicate the sweep direction of the magnetic field, negative to positive and vice versa.

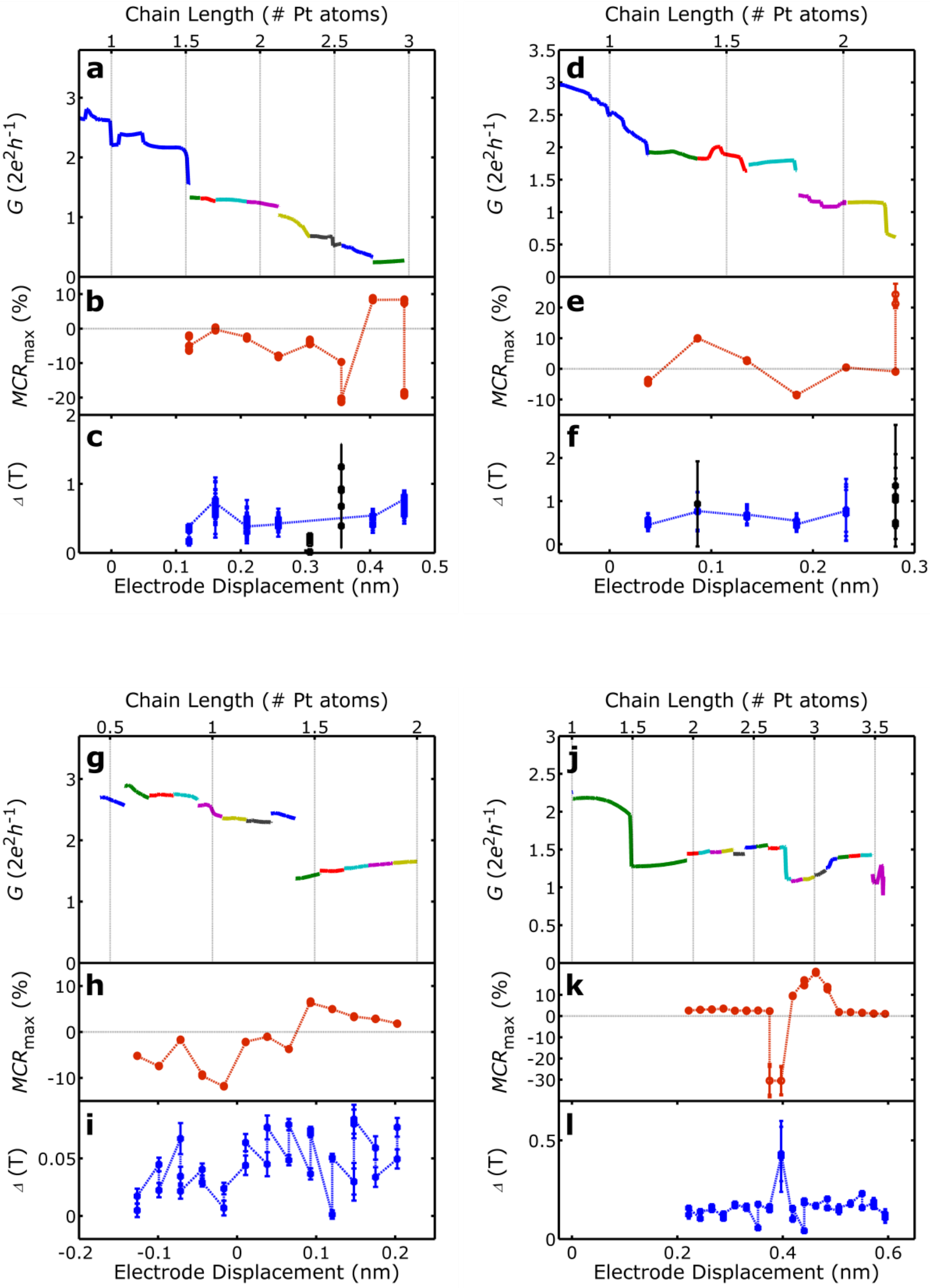

Supplementary Figure 2: Evolution of the conductance, maximum magnetoconductance ratio, and hysteresis Δ while breaking atomic Pt contacts. The motor was stepped in distances corresponding to 0.1 to 0.5 Å of electrode displacement in the atomic contact regime. On each step the MC was measured and the magneto-conductance amplitude MCR_{max} and Δ were determined from this data. The top axis shows the chain length in units of the distance of adjacent Pt atoms in atomic chains. The MCR_{max} shows values from -30% up to 25% varying from contact to contact. The hysteresis, ranging between 20 and 800 mT, not only depends on the individual contact, but also on the chain, within which the hysteresis shows a much smaller variation range. If the contact was not stable, or the fitting algorithm did not apply well, the hysteresis values were not taken into account for determining the hysteresis range; these values are marked as black symbols.

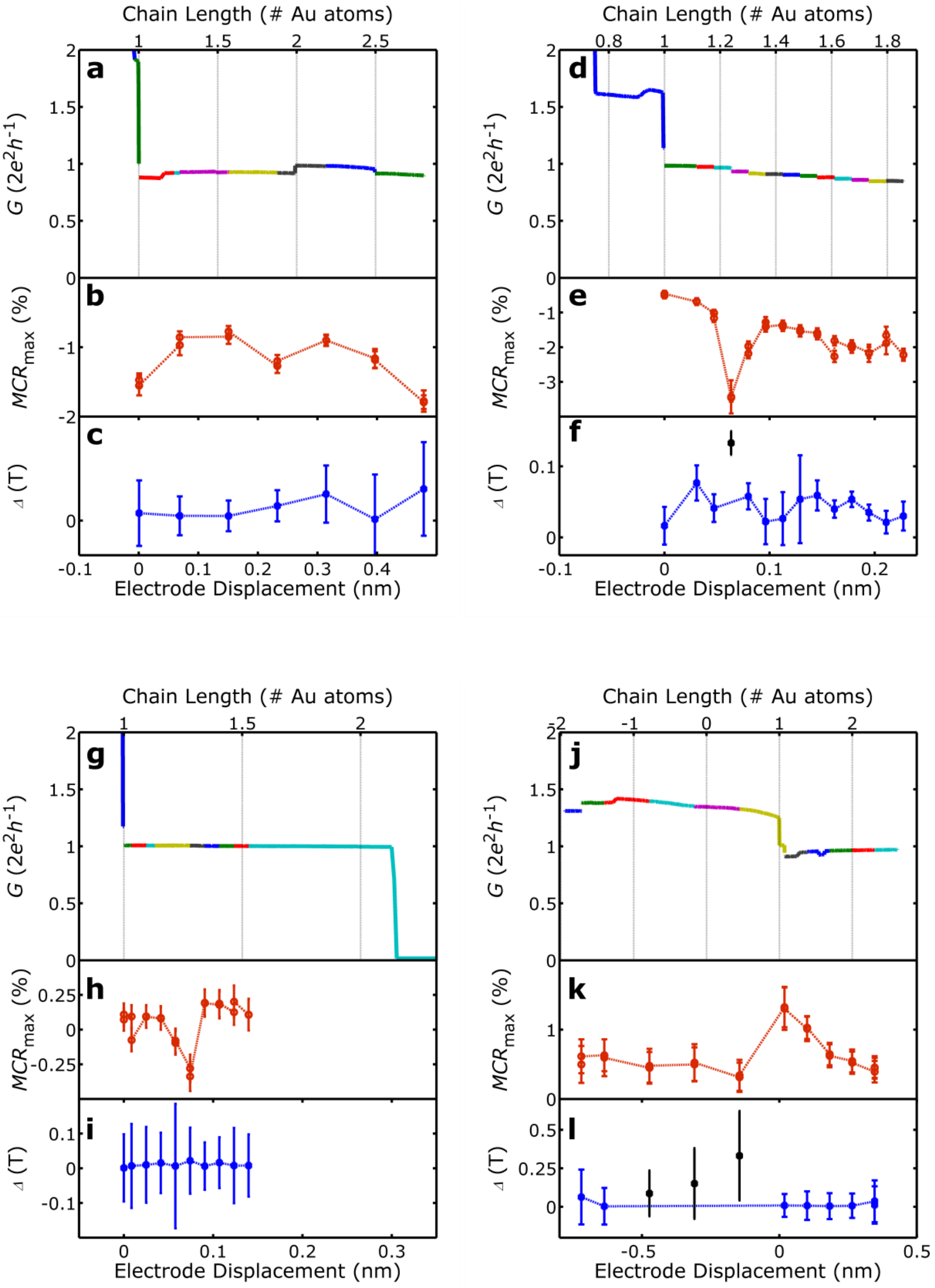

Supplementary Figure 3: A collection of opening traces of an Au sample. The motor was moved in steps corresponding to an electrode separation between 0.015 to 0.08 nm (see bottom axes) and for each step a magnetic field sweep was recorded. From these measurements the magneto-conductance ratio (MCR_{max}) and hysteresis Δ was calculated (left axes). In (**a**), (**b**) and (**c**) the motor stepping was started after getting to the single atomic contact (1 G_0), in (**d**) it was started on bigger contacts. The top axes show the electrode displacement in units of distance between adjacent Au atoms in an atomic chain (0.265 nm[5]), giving an estimation of how many atoms are present in the chain. Due to the small magneto-conductance (MC) amplitudes, which are dominated by conductance fluctuations, the hysteresis values have a huge uncertainty. For some contacts (black dots) the determination of the hysteresis value is not reliable, because the contact was unstable.

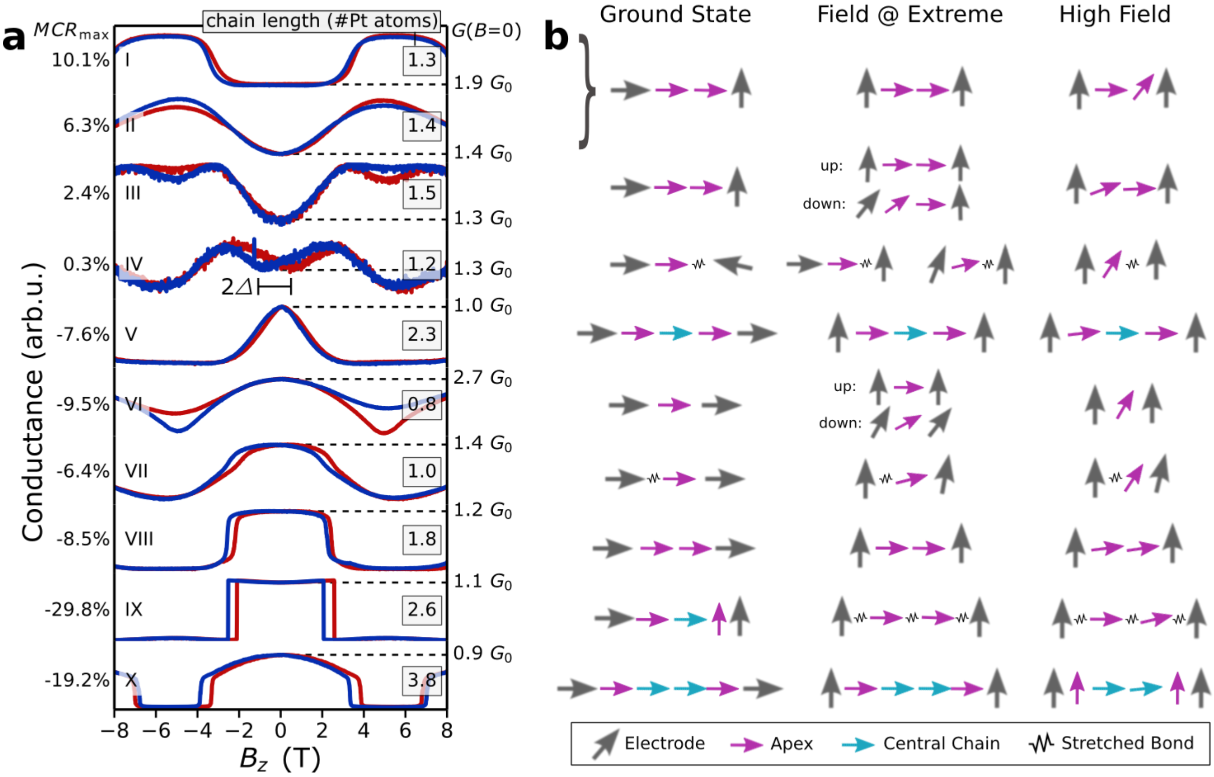

Supplementary Figure 4: Typical magneto-conductance curves and their interpretation. a Typical *MC* traces recorded in Pt atomic contacts and chains with different chain length up to four atoms (same data as Fig. 2a). **b** The sketches illustrate the interpretation of the magneto-conductance (MC) traces on the basis of our model, i.e. assuming that a misalignment among the magnetisation of the electrodes, the apex atoms and the chain will decrease the conductance of the contact. A possible configuration is shown for each curve at the ground state close to zero-field, the field corresponding to the extreme value of the *MCR* at $|\boldsymbol{B}| > 1$T as well as the high field region.

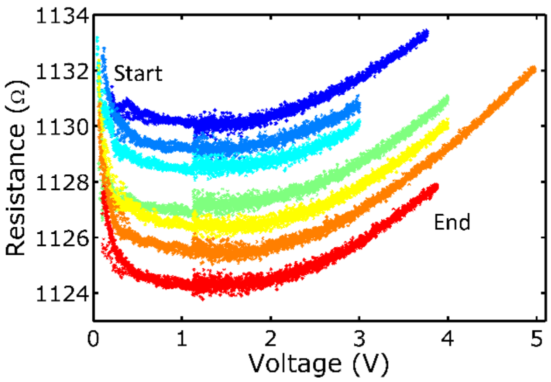

Supplementary Figure 5 | The resistance development of a Pt sample during electromigration. The presented data is raw data as extracted from the source measurement unit, without subtracting the serial resistance of the cryostat wiring, amounting approximately to 1000 Ω. The initial sample resistance thus amounts to roughly 130 Ω. Starting from this sample resistance, the resistance drops about one Ω per cycle, down to 123 Ω after six cycles, where the process was aborted. The maximum resistance change was adjusted to different values for a better process control. Please note the discontinuity at ~ 1.1 and 2.1 V are artefacts arising from range switching of the used source measure unit.

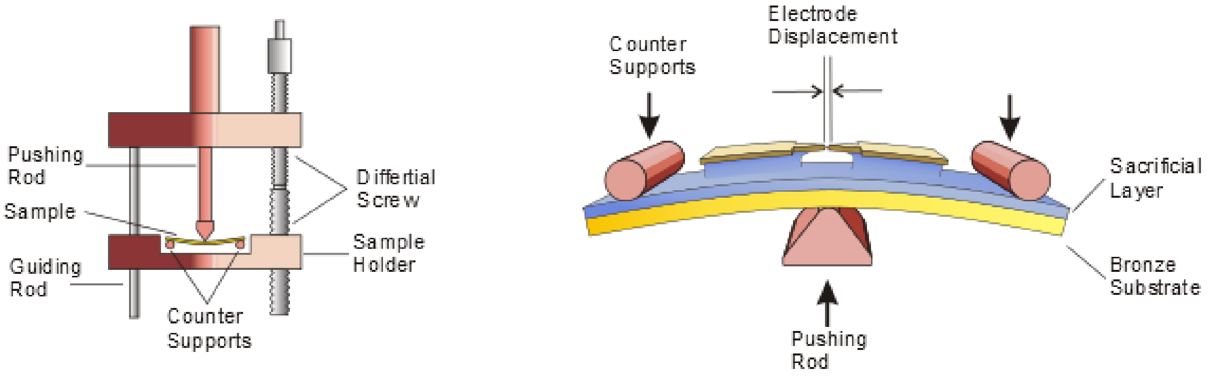

Supplementary Figure 6 | The breaking mechanism of a MCBJ setup. Left: By turning the differential screw, the pushing rod bends the substrate, thus leading to a strain in the sample which eventually breaks at the freestanding constriction. Right: The amount of electrode displacement induced by the pushing rod movement depends on the thickness of the substrate (here: 0.3 mm), the distance of the counter supports (here: 16 mm) and the freestanding length (here: 2 µm). With this setup design the resolution of electrode displacement is in range of a few picometres.

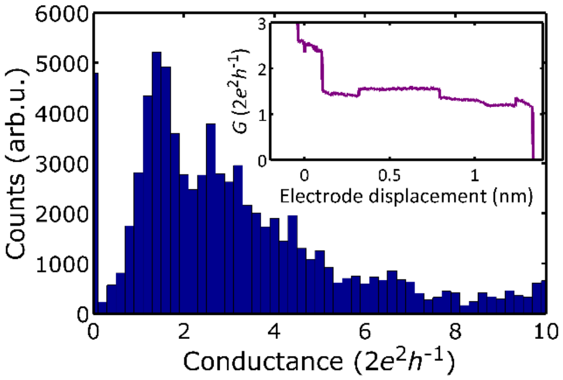

Supplementary Figure 7 | Conductance histogram of Pt. A typical conductance histogram of an electromigrated Pt MCBJ, calculated from 203 opening traces. The inset shows the last part of single opening trace recorded at 4.2 K. The conductance drops below 2.5 G_0, where the single atomic contact is formed[1]. The contact stays in the single atom regime for an electrode separation of ~1.3 nm. In atomic chains the distance of adjacent Pt atoms has been determined as 0.23 nm [1, 2, 3, 4]. With this value the chain has a length of 6 atoms at break.

Supplementary Tables

Supplementary Table 1 – Abundance of typical magneto-conductance traces recorded out of a total number of 165

Category	Absolute No.	Relative %
2b	53	32.1
2c	20	12.1
I / II	27	16.4
III	7	4.2
IV	9	5.5
V	2	1.2
VI/VII	31	18.8
VIII	5	3.0
IX	3	1.8
X	3	1.8

Supplementary Discussion

The two most common shapes are discussed in the main article and an interpretation is given there, see Fig. 2b, c. The major features of these "basic" forms are an extreme near zero and two extremes of inverse sign to this one at high magnetic fields. These are located almost symmetrically around zero-field in a range of 4-6T. The different amplitude of the high-field extremes between sweeping up and down the field is smaller than 0.5 %. Out of a totality of 165 curves that we analysed in detail, 73 (44%) fall into these two categories, 53 correspond to the type shown in Fig. 2b with a negative $MCR_{\max}$ value and 20 to Fig. 2c with positive $MCR_{\max}$. In the following we will discuss other typical curves also shown in Fig. 2a in the main article and Supplementary Figure 4a. While in many cases the region around the low- and high-field extremes follow a parabolic shape, we also regularly observe a plateau-like shape in these sections. The width of these plateaus is typically in the range of ~1T around the extremes, see curves I, III, V, and VII in Supplementary Figure 4a. In some cases the plateaus extend to the maximum field of ±8T and resemble a saturated state, see curves III, V, VIII. In other cases the height of the outer extremes, depends on the sweep direction. This difference can be small (curve II, and Fig. 2c), but also be in the range of several percent (curve VI). For several contacts we observe the occurrence of additional local extremes, see curves III and IV. These local extremes appear on all field scales and with varying sizes, indicating of a superposition of the basic shapes, or in other word a competition between contributions with similar amplitude and varying sign and field scale. The *MCR* of these shapes ranges from ~0.3% up to 10%.

We now give a tentative interpretation of the various curves (I-X in Supplementary Figure 4a) based on the simple model already used to describe the most abundant traces shown in Fig. 2b and 2c in the main article. The relative abundance of each type among 165 recorded MCR curves is given in Supplementary Table 1. Since the interpretation (see below) of curve-types VI, VII and I,II differ in small details only to those shown in Fig. 2c and 2b in the main article, one can group them in the same category. The main features of these curves are a continuous, non-monotonic MC with either positive (2c, I, II) or a negative (2b, VI, VII) sign and a hysteresis in the order of few hundred mT. As shown in Supplementary Table 1 the totality of curves belonging to these two categories account for approximately 80% of all curves. Moreover, curves with negative MC are far more common, by a factor of two, than those with positive MC requiring a non-collinear ground state. This is also in accordance with theoretical predictions that a non-collinearly magnetised ground state only occurs in rare circumstance. Most notably all, even the more unusual MC traces can be described following the same line of argumentation with the general assumption that a misalignment of the moments of chain and apex atoms as well as the electrodes with respect to each other leads to a decreased conductance. However, the relative amplitudes of these contributions as well as the additional influence of spin-orbit effect, such as the AMC due to a reorientation of moments in the constriction with respect to the current and possible changes of the bond-lengths cannot be evaluated so easily. Therefore the given interpretation of the individual *MCR* traces is only one of several other possibilities and may not be regarded as singular validity. Although similar curves than those indicated in Fig. 2a have been observed for different chain lengths, we restrict the discussion to the particular case shown in the figure. Another challenge for the interpretation is that the currently available calculations only consider chain lengths of three atoms or more. Since the perpendicular MAE of the apex atoms reported in Ref. [6] stems from the breaking of symmetry at the interface between chain and electrode, while the central atoms are in-plane magnetised due to shape anisotropy, one might conclude that for an atomic contact the central atom has similar characteristics as a chain atom. It is important to emphasise, that while pulling a chain the interatomic distance is subject to large variations[7], which is especially important for short chains. The bonding to one side of the electrodes will be preferred and lead to non-uniform bond lengths, giving rise to different MAE of the chain/apex atoms. For longer chains, the strain will be more equally distributed over all bonds in the chain. We treat these atoms in Supplementary Figure 4 and in the interpretation below as apex-atoms even though they may behave differently than in longer chains, where a strong coupling to the central atoms is present.

I and II: These curves are very similar to the one shown in Fig. 2c in the main article but have been measured for a rather short chain of 1.3 and 1.4 atoms nominal length. This length indicates that only one or two apex atoms are present in the contact across which the voltage drops locally. As for all traces showing a positive MC a possible scenario would be a ground-state with non-collinear magnetisation. When applying a field perpendicular to the chain direction the moments of both the electrode and eventually the apex atoms will be reoriented out of plane. In I, the absence of MC changes at low fields means that the magnetisation of the electrodes is not gradually tilted out of the plane but only after overcoming a threshold field of approximately ± 3T. This may be due to a strong coupling of the electrodes magnetisation to the apex atoms in an asymmetric contact. The same reason may apply for the decrease of the conductance upon tilting of the apex atoms out of plane. The conductance decrease at high fields $|\boldsymbol{B}| > 6$ T may also be explained by either orbital magnetoresistance or AMR. Both effects may give rise to positive MC in a similar order of magnitude. The curve in II shows a larger sweep-direction dependent conductance difference around the extremes at ± 5T. This effect is the main feature in magnetic tunnel junctions and is attributed there to the spin-polarisation of the electrodes and occurs there at smaller field scales, where an anti-parallel configuration of the electrodes is achieved. Since the field scale at which the differences are observed here corresponds to the reorientation fields of the apex atoms, we assume that here the conductance difference corresponds to the contribution by the relative misalignment between the two apex atoms, that depends on the sweep direction.

III: Curve III is similar to curve II but with smaller amplitude and with an additional minimum around 5 T (where in curve II a maximum occurs) when sweeping up the field, and at -5T when sweeping down. The following scenario would give rise to such behaviour: The ground state consists as in curve II of the chain, one electrode and the apex atoms magnetised in plane and the other electrode out of plane, but not necessarily perpendicular to the chain axis. When sweeping up the field, first the magnetisation of the electrodes align with the applied field and w.r.t. each other, enhancing G. Then, at higher fields one of the apex atom starts to reorient out-of-plane. However, the pinning of the apex atoms to the in-plane direction and their coupling to the electrodes is strong in such a short contact (here nominally 1.5 atoms). This coupling may also change with the magnetisation direction of the electrodes. As a result the apex atoms mainly remain magnetised in-plane but the orientation of the electrode's magnetisation differs after reversing the sweep direction, which then gives rise to a different conductance around ± 5T.

IV: Curve IV shows a typical shape found when the overall $MCR_{\max}$ is very small. It also starts with a minimum around zero-field and the high field extremes are minima as well. Also typical for this group of traces is a relatively high coercive field, indicating that the relative contribution of the electrodes is larger. The relation between the individual contributions due to the misalignment between the electrodes and the apex atom(s) and the spin-polarisation are difficult to predict without knowing the exact atomic arrangement and without a detailed theoretical model including spin-orbit scattering. If they are in the same order of magnitude they may eventually cancel each other resulting in rather small $MCR_{\max}$ with multiple extremes. Such behaviour has been found in approximately 5% of all recorded MC traces. The contact may be highly asymmetric, such that one of the electrodes couples strongly with the apex atom, while the other one is only weakly coupled. The large hysteresis then results from a different switching field of both electrodes.

V: Curve V is the first one featuring a maximum of the conductance at low field and a negative $MCR_{\max}$. However, despite the fact that a negative MCR is observed in the majority of cases, the particular type shown in curve V is quite rare (only 1.2% of all traces). The possible magnetisation configuration to explain this behaviour is probably similar to the one in Fig. 2b, where in the ground state all magnetisations are in plane. The weak increase of G above the high filed minimum, which was interpreted as arising from the misalignment between chain atoms and apex atoms, might indicate that here the apex atoms are stronger pinned to the central chain atom. This interpretation is supported

by the fact that curve IV has been recorded on a relatively long chain with a nominal chain length of 2.3 atoms.

VI: Curve VI is similar to the one of Fig. 2b in the main text but with pronounced conductance difference at the high-field extremes between the sweep directions. As in curve III the conductance differences indicates a different magnetisation state and coupling between the electrode and the apex atoms after reversing the sweep direction. Moreover, a strong contribution of the misalignment between apex atoms and the electrodes is expected for a short contact (here: 0.8 atoms) where the voltage drops locally

VII: Curve VII resembles the curve of Fig. 2b, but with a conductance plateau around zero-field, similar to curve I. The shape of the curve can be explained by a ground state with all magnetisations in plane. The decrease of the conductance starting around ± 1T shows the gradual tilting of the electrodes' magnetisations out of plane, and the increase above ±6 T indicates the tilting of the apex atom out of plane. Besides the plateau at low fields, the pronounced hysteresis in the up- and down sweeps also indicates a large coupling of the electrodes magnetisation to the apex atom and an asymmetric contact configuration as explained in V.

VIII: Curve VIII has a similar shape as Curve VII but with steeper slope of the conductance change around ±3T and weaker contribution above ±6T. A possible explanation would be a stronger spin-polarisation and stronger contribution of the electrodes to the MC. In the high-field region no clear extreme is observed; this indicates that the apex atoms are strongly pinned along the chain axis.

IX: Curve IX is an extreme variant of curve VIII with a step-like decrease of the conductance around ± 2.5 T and negligible increase at high fields. We exclusively observe these curves for chains that are close to a conductance change when stretched further. We therefore believe that these curves indicate reversible atomic rearrangements triggered by magnetic forces. It has been shown that in atomic contacts of bulk magnetic materials magnetostriction plays a crucial role[8]. Since in atomic Pt contacts, ferromagnetic order is limited to the contact area, these contributions are expected to be much smaller. Nevertheless we can attribute the observed conductance jumps to a reorganization of the contact geometry due to the remaining magnetostrictive forces and instabilities caused by the magnetic forces. Following Thiess *et al,* the formation of a chain is subject to a subtle interplay between the bonding strength of the chain atoms and the cohesive force in bulk[7]. Since a magnetization of the contact reduces the binding force between the atoms in the chain, an atom which is in the state of being extracted from bulk might snap back into the leads as a magnetic field overcomes a certain threshold[9]. This will leave the remaining chain with a higher bond length and thus a lower conductance[10], i.e. we expect negative MC as indeed observed for these particular configurations. Moreover, the atomic reconfiguration of the chain upon magnetisation could be accompanied by magnetic reorganisation of the contact. In Supplementary Figure 4b IX this is indicated by the in-plane alignment of the apex atom after at moderate fields > 3 T. As a result of this magnetic reconfiguration, the initially positive MC changes sign and a negative MC is observed at larger fields.

X: In addition to the large and steep conductance change at ±4T, curve X shows a step-up at high fields. Such step-like conductance features may indicate either abrupt changes of the orientation of the magnetisation or reversible atomic reconfigurations [7]. In X the MC of a long Pt chain of approximately four atoms is shown; in these chains the large pinning of chain and apex atoms along the chain axis as well as geometric instabilities can give rise to sudden conductance changes at certain threshold fields. From the analysis of a single curve at one temperature and fixed sampling rate these effects are difficult to distinguish.

Supplementary Methods

Sample characterisation

The conductance of the samples is measured after EM while carefully breaking the constriction. The opening traces, i.e. conductance vs. distance curves when stretching the nanobridge, reveal plateaus at certain conductance values, which indicate the existence of preferred atomic arrangements of the contact [1, 2]. The distribution of conductance data from a large number of breaking traces is compiled into a conductance histogram, i.e. a probability vs. conductance plot, which reveals peaks at the plateau values of the breaking traces [11, 12]. It has been shown that mechanical training by repeated breaking and forming contacts (by stretching and relaxing the bridge) very often (~10000 times) reduces the scatter on the plateaus and thus improves the visibility of the histogram peaks [13]. Since with our purely mechanical breaking mechanism, the breaking speed is limited to 0.1nm/s, this mechanical training is not efficient.

Nevertheless the conductance histogram which is recorded after EM shows a broad peak around 1.4 G_0 which is comparable to literature values [14] confirming the purity of the samples in the contact region, see Supplementary Figure 6.

Calibration of chain length

A characteristic property of Pt is the formation of monoatomic chains[11, 12]. This property stems from a relativistic effect, which ultimately leads to a reduced bond length and a higher bonding strength in low coordinate systems compared to bulk[1, 2].

To determine the atomic chain lengths, one of the first measurements for every sample is to calibrate the electrode displacement, by calculating the calibration factor between the movement of the pushing rod and the separation of the electrodes[15]. We define the reduction ratio as the induced electrode displacement divided by the movement of the pushing rod.

To this end we measure the development of the tunnelling current, which depends exponentially on the size of the vacuum tunnel gap.

$$I \sim V \cdot \exp\left(-\frac{2}{\hbar}\sqrt{2m_{eff}\phi} \cdot D\right) \qquad (1)$$

Here m_{eff} denotes the effective electron mass (Pt: 2.6 m_e, Au: 1.1 m_e), ϕ the work function and D is the electrode distance. From this relation we obtain a dependence of the conductance on the distance of the electrodes.

$$\frac{d}{dD}\ln(G) \sim -\frac{2}{\hbar}\sqrt{2m_{eff}\phi} \qquad (2)$$

Different values for the work functions have been reported [16, 17]. We chose 5.65 eV for Pt and 5.1 eV for Au, both values yield an upper boundary, which leads to a slight underestimation of the reduction ratio and hence to a lower bound in the estimation of chain length.

We measure the conductance in the tunnelling regime while closing the sample over several orders of magnitude and fit the curve with an exponential function, as described in Eq. 2 for obtaining the reduction ratio. For our samples we obtain reduction ratios of 1:19000 to 1:30000 which is in agreement with estimations from the geometry and previous works[1, 2, 11, 12, 15]. The error for the reduction ratio as well as for the resulting chain length is about 30%.

A general aspect when forming atomic chains by pulling apart macroscopic electrodes is that the interatomic distance is not controlled. For lithographic break-junctions as used here the problem is less pronounced than for STMs or break-junctions formed from notched, macroscopic wires because the freestanding length is much shorter and therefore elastic expansion of the electrodes are suppressed. Nevertheless with notched-wire break-junctions and with STMs it has been shown that statistically the wire length of atomic chains corresponds to multiples of atom diameters with somewhat reduced

interatomic distance[1, 12], suggesting that the bonds between atoms are stretched homogeneously. We also use this assumption when indicating the chain length in numbers of atoms, which is calculated from the electrode separation, estimated from the tunnelling distance-dependence and a fixed bond length for Pt (0.23 nm) [3, 4].

Supplementary References

1. Smit R, Untiedt C, Yanson A, van Ruitenbeek J. Common Origin for Surface Reconstruction and the Formation of Chains of Metal Atoms. *Physical Review Letters* **87**, 266102 (2001).

2. Bahn S, Jacobsen K. Chain Formation of Metal Atoms. *Physical Review Letters* **87**, 266101 (2001).

3. Pauly F, Dreher M, Viljas J, Häfner M, Cuevas J, Nielaba P. Theoretical analysis of the conductance histograms and structural properties of Ag, Pt, and Ni nanocontacts. *Physical Review B* **74**, 235106 (2006).

4. Smogunov A, Dal Corso A, Tosatti E. Magnetic phenomena, spin-orbit effects, and Landauer conductance in Pt nanowire contacts: Density-functional theory calculations. *Physical Review B* **78**, 014423 (2008).

5. Tung JC, Guo GY. Magnetic moment and magnetic anisotropy of linear and zigzag 4d and 5d transition metal nanowires: First-principles calculations. *Physical Review B* **81**, 094422 (2010).

6. Thiess A, Mokrousov Y, Heinze S. Competing magnetic anisotropies in atomic-scale junctions. *Physical Review B* **81**, 054433 (2010).

7. Thiess A, Mokrousov Y, Blügel S, Heinze S. Theory and Application of Chain Formation in Break Junctions. *Nano Letters* **8**, 2144-2149 (2008).

8. Egle S*, et al.* Magnetoresistance of atomic-size contacts realized with mechanically controllable break junctions. *Physical Review B* **81**, 134402 (2010).

9. Thiess A, Mokrousov Y, Heinze S, Blügel S. Magnetically Hindered Chain Formation in Transition-Metal Break Junctions. *Physical Review Letters* **103**, 217201 (2009).

10. Cuevas JC, Yeyati AL, Martin-Rodero A, Rubio Bollinger G, Untiedt C, Agraït N. Evolution of Conducting Channels in Metallic Atomic Contacts under Elastic Deformation. *Physical Review Letters* **81**, 2990-2993 (1998).

11. Yanson AI, Rubio Bollinger G, van den Brom HE, Agraït N, van Ruitenbeek JM. Formation and manipulation of a metallic wire of single gold atoms. *Nature* **395**, 783-785 (1998).

12. Agraït N, Yeyati AL, van Ruitenbeek JM. Quantum properties of atomic-sized conductors. *Physics Reports* **377**, 81-279 (2003).

13. Sabater C, Untiedt C, Palacios JJ, Caturla MJ. Mechanical Annealing of Metallic Electrodes at the Atomic Scale. *Physical Review Letters* **108**, 205502 (2012).

14. Nielsen S*, et al.* Conductance of single-atom platinum contacts: Voltage dependence of the conductance histogram. *Physical Review B* **67**, 245411 (2003).

15. van Ruitenbeek JM*, et al.* Adjustable nanofabricated atomic size contacts. *Review of Scientific Instruments* **67**, 108-111 (1996).

16. Fomenko VS, Samsonov GV. Handbook of Thermionic Properties. 5-68 (1966).

17. Michaelson HB. The work function of the elements and its periodicity. *Journal of Applied Physics* **48**, 4729-4733 (1977).
